# Evaluation of Cardiovascular Risk Factors in the Wistar Audiogenic Rat (WAR) Strain

**DOI:** 10.1371/journal.pone.0129574

**Published:** 2015-06-01

**Authors:** Rubens Fazan, Carlos Alberto A. Silva, José Antônio Cortes Oliveira, Helio Cesar Salgado, Nicola Montano, Norberto Garcia-Cairasco

**Affiliations:** 1 Department of Physiology, School of Medicine of Ribeirão Preto, University of São Paulo, Ribeirão Preto, São Paulo, Brazil; 2 Department of Clinical Sciences “Luigi Sacco”, University of Milan, Milan, Italy; University of Buenos Aires, Faculty of Medicine. Cardiovascular Pathophysiology Institute., ARGENTINA

## Abstract

**Introduction:**

Risk factors for life-threatening cardiovascular events were evaluated in an experimental model of epilepsy, the Wistar Audiogenic Rat (WAR) strain.

**Methods:**

We used long-term ECG recordings in conscious, one year old, WAR and Wistar control counterparts to evaluate spontaneous arrhythmias and heart rate variability, a tool to assess autonomic cardiac control. Ventricular function was also evaluated using the pressure-volume conductance system in anesthetized rats.

**Results:**

Basal RR interval (RRi) was similar between WAR and Wistar rats (188±5 vs 199±6 ms). RRi variability strongly suggests that WAR present an autonomic imbalance with sympathetic overactivity, which is an isolated risk factor for cardiovascular events. Anesthetized WAR showed lower arterial pressure (92±3 vs 115±5 mmHg) and exhibited indices of systolic dysfunction, such as higher ventricle end-diastolic pressure (9.2±0.6 vs 5.6±1 mmHg) and volume (137±9 vs 68±9 μL) as well as lower rate of increase in ventricular pressure (5266±602 vs 7320±538 mmHg.s-1). Indices of diastolic cardiac function, such as lower rate of decrease in ventricular pressure (-5014±780 vs -7766±998 mmHg.s^-1^) and a higher slope of the linear relationship between end-diastolic pressure and volume (0.078±0.011 vs 0.036±0.011 mmHg.μL), were also found in WAR as compared to Wistar control rats. Moreover, Wistar rats had 3 to 6 ventricular ectopic beats, whereas WAR showed 15 to 30 ectopic beats out of the 20,000 beats analyzed in each rat.

**Conclusions:**

The autonomic imbalance observed previously at younger age is also present in aged WAR and, additionally, a cardiac dysfunction was also observed in the rats. These findings make this experimental model of epilepsy a valuable tool to study risk factors for cardiovascular events in epilepsy.

## Introduction

Epidemiological studies have established that the incidence of premature death among epileptic patients is markedly higher than in the general population [1,2,3]. There has been growing awareness of sudden unexpected death in epilepsy (SUDEP), which is now acknowledged as a serious problem for epileptic patients [2,3]. Evidence from epidemiological, clinical, and pathological studies indicates that in most cases SUDEP occurs after a seizure, although deaths not preceded by seizure have been reported anecdotally [3].

Even though the pathophysiology of SUDEP is unknown in most cases, it is important to consider that sudden death is often attributed to cardiac events. Heart dysfunction and disturbances in neural control of the cardiovascular system are always associated with morbidity and mortality in epileptic patients [3,4,5,6]. In fact, QT dispersion, reflecting regional heterogeneity of cardiac repolarization and important risk factors for ventricular arrhythmias, have been seen in up to one third of people with epilepsy [7,8]. Moreover, few studies with electrocardiographic (ECG) recordings during or near to sudden deaths in epileptic patients suggest that cardiac arrhythmias are crucial determinants of some of them [9,10].

Clinical and experimental studies provide evidences that epileptic seizures are accompanied by autonomic cardiovascular imbalance with sympathetic overactivity, resulting in hypertension, tachycardia and electrical instability in the heart [10,11,12]. Although changes in sympathetic discharges are known to occur during epileptic seizures, autonomic disturbances, mostly showing enhanced sympathetic cardiovascular tone, were also found in inter-ictal periods in either epileptic patients [13, 14,15,16,17] or experimental models of epilepsy [18].

It has long been recognized that increased sympathetic activity has a profound influence on the electrical and contractile functions of the heart [19]. There is wide evidence that excessive catecholamine release, due to sympathetic activation, produces severe toxic cardiac effects, such as intra-cellular Ca^2+^ overload, high-energy phosphate depletion, life threatening ventricular arrhythmias, and sudden cardiac death [19,20,21,22]. Moreover, long-lasting sympathetic overactivity, known to promote myocardial apoptosis [23] and cardiac hypertrophy with chamber remodeling [24,25] is thought to be a major determinant of progressive heart failure.

Remarkable advances in the understanding of autonomic function have been made due to the development of methods to evaluate short-term changes in heart rate (HR) and/or blood pressure (BP). The notion that, in addition to cardiac cycle, other rhythms related to sympathetic and parasympathetic drives to the heart are present in BP and HR series stimulated great interest in using measures of cardiovascular variability as diagnostic tools [26]. Analysis of spontaneous rhythmic fluctuations in HR by spectral analysis offers remarkable insight into the physiological mechanisms of autonomic cardiovascular control [27,28,29,30,31].

Various experimental animal models of epilepsy have been used to examine the pathophysiological mechanisms of this syndrome. For example, Wistar audiogenic rats (WAR) [32,33], which are an inbred strain prone to audiogenic epileptic seizures derived from Wistar rats, have been used extensively as an experimental model of epilepsy. A recent study from our laboratory [18] showed that young adult WAR are slightly hypertensive, have higher basal HR and a 2-fold higher cardiac sympathetic tone compared with Wistar control rats with no experience of audiogenic epileptic seizures.

It is well established that high BP and chronic cardiac sympathetic overactivity are leading causes of life-threatening cardiovascular events, such as ventricular arrhythmias and are also major determinates of heart failure [23,24,25]. In this context we hypothesize that WAR are at higher cardiovascular risk, especially during aging. Therefore, the aim of this study was to identify spontaneous arrhythmias, as well as to evaluate HR and BP variability as an approach to characterize autonomic modulation of the cardiovascular system in aged, conscious freely-moving WAR. Moreover, cardiac function was also evaluated by analyzing left ventricular pressure-volume relationships in anesthetized rats from the WAR strain.

## Methods

### Ethics Statement

All procedures were performed in accordance with institutional polices and guidelines for ethical use of animals and following the recommendations from the Brazilian Society for Neuroscience and Behavior. At the end of the ventricular pressure recordings, with the animals still under the effect of anesthesia (see below), by mean of large laparotomy, they were killed by ample diaphragm opening. Specific protocols for the current experiments were approved by the Commission for Animal Experimentation (CETEA; 117/2011 and 16/2013-1) of the Ribeirão Preto School of Medicine.

The experiments were conducted on male WAR and their Wistar counterparts (control, n = 9 in each group) at 11 to 12 months of age with no prior induction of audiogenic epileptic seizure (WAR) and sound stimulation (Wistar). The animals were housed individually with free access to food and water and were maintained on a 12/12 h light/dark cycle at 22°C.

### Surgery and Recording of Electrocardiogram

The animals were anesthetized with tribromoethanol (250 mg/kg i.p.) and implanted with subcutaneous ECG electrodes two days prior to recording. The electrodes tips were suture-fixed subcutaneously and the wires exteriorized at the back of the neck and the rats were allowed to recover for 2 days. The animals were taken to the recording room, where the electrodes were connected to an ECG amplifier (6600 series ECG/Biothac. Amplifier, Gould Instrument Systems In, Valley View, OH, USA) coupled to an A/D interface (DI720 Dataq Instruments Akron, OH, USA) at least 30 min before beginning the recordings. The room was kept at 22±1°C, and silence was maintained to minimize environmental stress. After the adaptation period the ECG was digitally recorded (2 kHz) for 60 min.

ECG segments with 20,000 beats were analyzed using the computer software ECG module for LabChart 7.2 (ADInstruments, Mountain View, CA, USA) that automatically detected normal and ventricular premature beats (VPB).

### Cardiac Function

At the end of the ECG recording, the animals were anesthetized with sodium pentobarbital (40 mg/kg, ip) and placed on a controlled heating pad to maintain the core body temperature at 37°C. A microtip pressure-volume catheter (SPR-838, Millar Instruments, Houston, TX, USA) was placed into the left ventricle through right carotid artery as described elsewhere [34]. After stabilization, the ventricular pressure and volume signals were continuously recorded using the pressure-volume conductance system (MPVS, Millar Instruments, Houston, TX, USA) coupled to a PowerLab/4SP (AD Instruments, Mountain View, CA, USA). The HR, ventricular end-diastolic pressure (EDP), maximal slopes of the systolic pressure increment (+dP/dt_max) and diastolic pressure decrement (-dP/dt_max), relaxation time constant (), ejection fraction (EF), end-systolic and end-diastolic volumes (ESV and EDV) as well as cardiac output (CO) were calculated using a cardiac pressure-volume analysis software (PVAN Ultra v3.2, Millar Instruments, Houston, TX, USA) [34]. The total peripheral resistance (TPR) was also calculated as the ratio between MBP and CO. A range of left ventricle pressure-volume curves at different pre-load status was assessed by transiently compressing the inferior vena cava. Pre-load independent indices of heart contractility and stiffness {preload recruitable stroke work (PRSW), +dP/dt vs. end-diastolic volume relationship (+dP/dt-EDV), slopes of end-systolic and end-diastolic pressure-volume relations [ESPVR and EDPVR]} as well as the efficiency of left ventricle work [pressure-volume area] were calculated using PVAN v3.2. Good quality recordings of ventricular pressure and volume were obtained in only 7 WAR and 8 Wistar rats.

### Heart Rate Variability

Files with ECG recordings were analyzed *off line* using the computer software LabChart 7.2 (AD Instruments, Mountain View, CA, USA). R waves were identified every cardiac beat and series of successive RR intervals (RRi) were generated for each animal. Over 5 to 10 stationary RRi series of 500±100 beats were selected by visual inspection of the entire recording.

After calculation of standard deviation of successive normal RRi (SDNN), an index of overall HRV (time domain) each segment spectral analysis using an autoregressive algorithm with the model order chosen according to Akaike’s criterion to obtain the frequency spectrum of HRV [28,31]. The oscillations of RRi were quantified in 3 frequency bands according to the central frequency of each component modeled by autoregressive algorithm: very low (VLF, below 0.2 Hz), low (LF, 0.2–0.8 Hz) and high frequency (HF, 0.8–2.0 Hz). The LF and HF oscillatory components were expressed in absolute (ms^2^) and normalized units as well as a ratio of low to high frequency (LF/HF). Normalization consisted of dividing the power of a given spectral component by the total power minus the power below 0.2 Hz and multiplying the ratio by 100 [29,35].

### Statistical Analysis

Each parameter, i.e. heart rate variability and cardiac function indexes, was firstly tested for normality of the distribution by modified Kolmogorov-Smirnov test (goodness of fit test) Following, they were compared between WAR and Wistar control counterparts by 2-tail unpaired Student t test or Mann-Whitney test, accordingly. A value of p< 0.05 was considered statistically significant. The data are presented as the mean±SEM.

## Results

The basal RR interval (RRi) was similar between WAR (188±5 ms) and Wistar control counterparts (199±6 ms).

The mean levels, SDNN and power of oscillatory components (autoregressive spectral analysis) of RRi during baseline recordings from both groups of rats are shown in Table 1. While the mean RRi (SDNN) did not differ between the groups, WAR showed higher overall RRi variability. Moreover, the normalized LF and the LF/HF ratio were markedly increased in WAR compared to Wistar counterparts.

**Table 1:** Mean values of RR interval (RRi) and RRi variability in time and frequency domain in one-year old conscious freely moving WAR and Wistar control rats.

	Control (Wistar)	WAR	P
**RRi (ms)**	199 ± 6	188 ± 5	0.1199
**SDNN (ms)**	4.8 ± 0.5	9.6 ± 1.2 *	0.0013
**VLF (ms** ^**2**^ **)**	20.7 ± 2.8	56.2 ± 9.9 *	0.0019
**LF (ms** ^**2**^ **)**	0.9 ± 0.3	10.5 ± 1.6 *	<0.001
**HF (ms** ^**2**^ **)**	11.0 ± 3.5	42.5 ± 6.9 *	0.0067
**LF (nu)**	7 ± 1	20 ± 1 *	<0.001
**HF (nu)**	93 ± 1	80 ± 1 *	<0.001
**LF (Hz)**	0.43 ± 0.07	0.45 ± 0.02	0.8048
**HF (Hz)**	1.67 ± 0.06	1.57 ± 0.04	0.2239
**LF/HF**	0.076±0.013	0.245±0.013 *	<0.001

*P<0.05 compared with Wistar control rats.

RRi: interval between successive R waves, SDNN: standard deviation of normal RRi, VLF, LF and HF: power of oscillatory components of RRi series at very low (<0.2 Hz), low (0.2–0.8 Hz) and high (0.8–3.0 Hz) frequency bands. nu: normalized units. LF and HF (Hz): central frequency of the main oscillatory component modeled at low- and high-frequency bands.

Values are mean ± SEM.

Hemodynamic parameters and indices of cardiac function derived from the left ventricle pressure-volume relationship in pentobarbital-anesthetized rats are shown in Table 2. HR did not differ between the groups while mean BP (MBP) and ventricular systolic pressure (LVSP, mmHg) were lower in WAR than in Wistar control rats. WAR also showed indices of systolic dysfunction, such as higher ventricular end-diastolic pressure (LVEDP, mmHg), lower +dP/dt_max (mmHg.s^-1^). Indices of diastolic cardiac function were also altered in WAR compared with Wistar control rats.-dP/dt_max (mmHg.s^-1^) was smaller in WAR, while time constant of ventricular diastolic pressure decay (tau, ms) and slope of the linear relationship between end-diastolic pressure and end-diastolic volume (EDPVR, mmHg.μL) were both higher in WAR as compared to Wistar rats.

**Table 2:** Hemodynamic parameters and indices of systolic and diastolic function derived from left ventricle pressure-volume relationship in one-year old pentobarbital anesthetized WAR and Wistar control rats.

	Control (Wistar)	WAR	P
***Parameters***			
HR (bpm)	322±14	292±15	0.1829
MBP(mmHg)	115±5	92±3 *	0.0008
LVSP (mmHg)	135±4	103±4 *	0.0001
ESV (μL)	68±9	137±9 *	0.0002
EDP (mmHg)	5.6±1.0	9.2±0.6 *	0.0161
EDV (μL)	154±11	210±11	0.0030
CO (mL.min^-1^)	28.2±4.3	21.4±2.3	0.1764
TPR (mmHg.mL^-1^)	4.5±0.6	4.6±0.4	0.8955
***Systolic Indexes***			
EF (%)	56±5	35±2 *	0.0016
+dP/dt_max (mmHg.s^-1^. min)	7320±538	5266±620 *	0.0283
ESPVR slope (mmHg.μL^-1^)	2.6±0.4	1.9±0.1	0.1179
PRSW (mmHg)	49±6	36±4	0.0765
***Diastolic Indexes***			
-dP/dt_max (mmHg.s^-1^)	-7766±998	-5014±780 *	0.0463
**τ** (ms)	15.1±0.5	21.8±2.9	0.0531
EDPVR slope (mmHg.μL^-1^)	0.036±0.011	0.078±0.011 *	0.0186

*P<0.05 compared with Wistar control rats.

HR: heart rate, MAP: mean arterial pressure, LVSP: left ventricular systolic pressure, ESV: end-systolic volume, EDP: end-diastolic pressure, EDV: end-diastolic volume, CO: cardiac output, TPR: total peripheral resistance, EF: ejection fraction, dP/dt_max maximal slopes of the systolic pressure increment (+dP/dt_max) and diastolic pressure decrement (-dP/dt_max), ESPVR and EDPVR: slope of end-systolic and end-diastolic pressure-volume relationships, τ: relaxation time constant, PRSW: preload recruitable stroke work.

Values are mean ± SEM.

**Fig 1:**
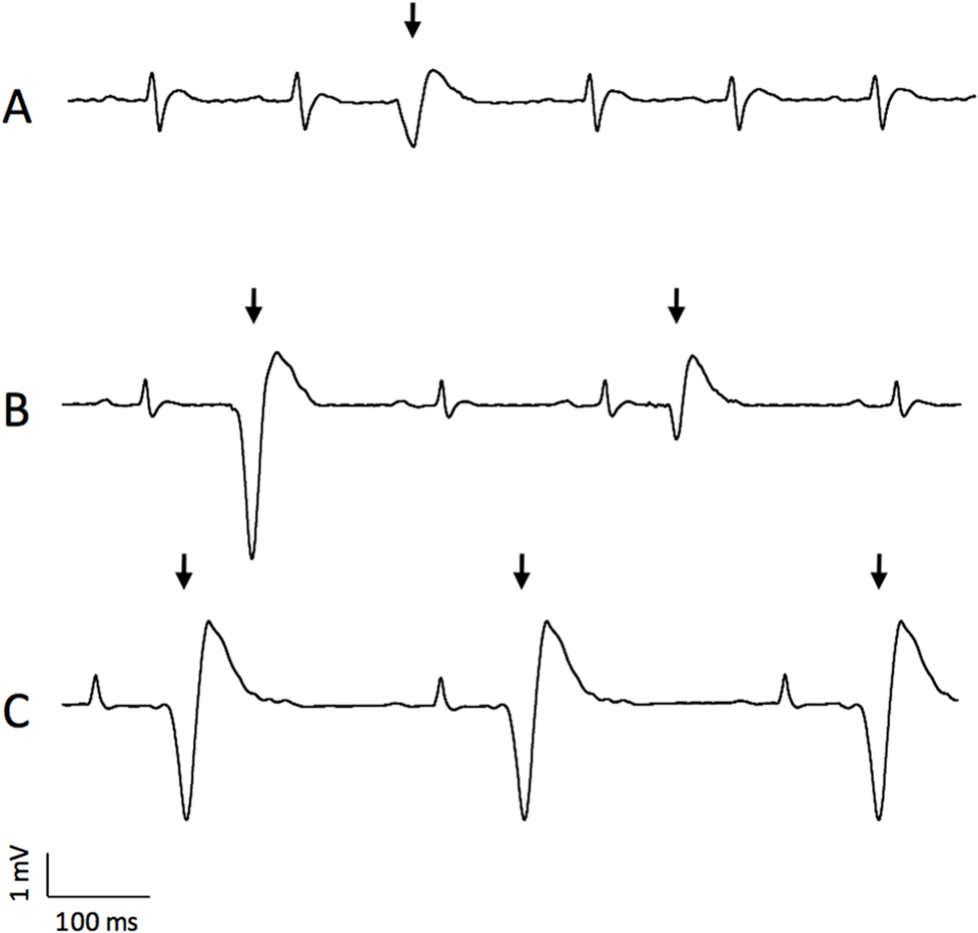
ECG recordings illustrating ectopic beats (arrows) in 3 different WAR studied at 1 year of age. (A) One isolated ectopic beat, (B) two ectopic beats from distinct ventricular origins and (C) a period of bigeminy.

Only 3 out of 9 Wistar control rats exhibited VPB in a range of 3 to 6 premature beats among the 20,000 beats analyzed in each rat (0.1±0.003 ectopic beats/1,000 normal beats). On the other hand, 6 out of 8 WAR showed arrhythmic beats ranging from 15 to 32 VPB out of the 20,000 beats evaluated (0.41±0.2 ectopic beats/1,000 normal beats; P<0.001, compared with Wistar counterparts). All VPB observed for Wistar control rats came from a single ectopic ventricular origin, whereas 4 of 9 WAR studied showed VPB from multiple ventricular origins. Moreover, a short period (approximately 1.5 s) of bigeminy was observed in one WAR, as illustrated in Fig 1.

## Discussion

We evaluated highly relevant parameters commonly used to determine risk for life-threatening cardiovascular events and sudden death, such as autonomic cardiac modulation, arrhythmias and cardiac function. The study was performed in aged WAR, which is a well-established experimental model of epileptic seizures. HR variability was performed in conscious freely moving animals instrumented with ECG electrodes. Surgical stress for subcutaneous implant of electrodes is minimal and since both groups were treated similarly no bias due to surgical recovery should affect the results. A recent study from our laboratory [18] showed that young adult WAR (70 days) present with mild hypertension and an autonomic cardiac imbalance, with sympathetic predominance. Moreover, the hypothalamus-pituitary-adrenal axis of WAR was previously found to be altered [36], which is consistent with the higher sympathetic drive of these animals and with the anxious profile of WAR in the elevated plus maze and in the open field [37]. These findings led to the use of older rats (11–12 months) in the present study, once it is widely accepted that the incidence of cardiovascular disorders associated with sympathetic imbalance, markedly increases with aging [38,39,40]. In addition, chronic sympathetic overactivity is highly recognized as one of the most important determinants in the development of age-related heart failure, which is an isolated risk factor for cardiovascular events, including sudden death [23,24,25].

Differently to our previous study [18], resting HR was found similar between WAR and aged match Wistar control rats. Two striking differences in the protocols used in both studies can be highlighted and are certainly involved in this discrepancy. First, the age of the rats evaluated in both studies. The function of sinoatrial node, the pacemaker of the heart, is known to decline during the ageing process, [41]. Moreover, a loss of autonomic influence to the heart is also associated with ageing [42]. Therefore, the use of aged animals in the present study might explain the similar HR between WAR and Wistar control rats. Second, in our previous study [18] HR was calculated from pulsatile BP recorded from a catheter placed in the femoral artery 24 h prior to the experiment. In the present study, subcutaneous ECG electrodes, placed 48 h before recordings, were used to calculate HR.

WAR exhibited greater overall RRi variability compared with aged-matched control Wistar rats. The spectra of RRi, when calculated in absolute units (ms^2^), also revealed higher power at both, LF and HF bands in WAR. These findings support the idea of a higher cardiac autonomic drive in WAR, i.e. greater sympathetic and vagal cardiac modulation. Nevertheless, LF power of RRi spectra was remarkably higher in WAR (10-fold) as compared to Wistar counterparts, leading to a higher LF and lower HF power of RRi spectra when expressed in normalized units. Thence, the LF/HF ratio, a well-accepted index of sympatho-vagal balance [28,29,35,43] was notably higher in WAR when compared with control rats. Power of PI spectra at LF band is widely accepted as an index of sympathetic modulation, while HF band power is associated with parasympathetic modulation [28,29,31]. Nevertheless, there is evidence that the LF power of HR spectra also correlates with parasympathetic modulation [43]. Thus, the powers of LF and HF bands more accurately represent autonomic modulation when presented in normalized units or as an LF/HF ratio [28,29,35].

Therefore, RRi variability evaluated in the present study in aged WAR strongly suggests an autonomic imbalance with sympathetic overactivity in this strain of aged rats. Substantial clinical and experimental evidence links sustained sympathetic activation to increased risk of life-threatening cardiovascular events, especially ventricular arrhythmias [38,44]. Furthermore, there is evidence that the most significant and widely-accepted mechanism of SUDEP involves cardiac arrhythmia induced by seizure discharges acting through the autonomic nervous system [3,45]. In line with our findings in WAR, there are evidences that epileptic patients also present interictal abnormalities in the autonomic modulation of cardiac activity, with high sympathetic and impaired vagal tone to the heart [14,15,16]. Changes in parasympathetic HR modulation have been noticed in either temporal lobe [13,46] or generalized epilepsies [47,48]. Moreover, anti-epileptic therapy, specifically with carbamezapine may also alter autonomic functions, increasing cardiovascular risk in epileptic patients [13,49].

The balance between sympathetic and vagal influences on the heart is a major regulator of cardiac function and can be a major cause of cardiac dysfunction. Diseases such as myocardial infarction, congestive heart failure and coronary disease are commonly associated with alterations in the normal sympatho-vagal balance [50].

To our knowledge, this is the first study to evaluate cardiac function in an experimental epilepsy model using a pressure-volume conductance system, a unique and powerful approach to evaluating systolic and diastolic function *in vivo* [34]. Young (70 days) WAR recorded in conscious freely moving state showed a mild hypertension [18] while aged (1 one year old) WAR, recorded under pentobarbital anesthesia, presented lower levels of basal BP as compared to Wistar control counterparts. The age of the animals, but mainly the effect of anesthesia should explain the difference in BP between these two studies. Aged WAR showed impaired systolic performance accompanied by delayed relaxation and increased diastolic stiffness of the left ventricle.

We found a lower +dP/dt_max and EF in aged WAR. Although +dP/dt_max has historically been used as a cardiac contractile index, this parameter is known to be load-dependent (especially preload-dependent) [51,52,53]. EF is also acknowledged to be influenced by both preload and afterload and cannot be used reliably to assess systolic function in models with changes in preload and/or afterload. The slope of ESPVR was proposed as a relatively load-insensitive index of ventricle contractility [51,54]. In the present study, ESPVR was decreased in WAR compared with Wistar rats. TPRI did not differ between the groups.

The data for the end-diastolic and end-systolic pressure volume relationships are presented in Table 2. The ESPVR slope was steeper in Wistar control rats than in WAR, suggesting decreased systolic performance in WAR. Moreover, EDPVR was increased in WAR compared to controls, indicating increased myocardial stiffness in WAR.

Impaired ventricular relaxation and increased end-diastolic stiffness were also observed in WAR, as reflected in the decreased-dP/dt_max, prolonged Tau, and increased LVEDP and EDPVR seen in these animals. Relaxation is an active process, depending mostly on calcium uptake during the diastole. End-diastolic stiffness is predominantly affected by changes in myocardial structural components [55].

A recent review article from Finsterer and Wahbi [56] regarding neurological diseases affecting the heart, describes that epilepsy is rarely directly associated with heart failure. Nevertheless, since this disease in either humans or experimental animals is often associated with high blood pressure and autonomic disturbances, the risk of the development of heart failure during aging is expected to be markedly higher in subjects with epilepsy.

Further studies will be extremely important for understanding the mechanisms of autonomic imbalance in WAR, for identifying risk factors and triggers of cardiovascular events related to epileptic seizures, and for critically assessing potential strategies to minimize those risks.
